# Comparison of regional vs. general anesthesia on the risk of dementia: a systematic review and meta-analysis

**DOI:** 10.3389/fpubh.2024.1362461

**Published:** 2024-06-03

**Authors:** I-Wen Chen, Cheuk-Kwan Sun, Jen-Yin Chen, Hsiao-Tien Chen, Kuo-Mao Lan, Kuo-Chuan Hung, Ching-Chung Ko

**Affiliations:** ^1^Department of Anesthesiology, Chi Mei Medical Center, Liouying, Tainan City, Taiwan; ^2^Department of Emergency Medicine, E-Da Dachang Hospital, I-Shou University, Kaohsiung City, Taiwan; ^3^School of Medicine for International Students, College of Medicine, I-Shou University, Kaohsiung City, Taiwan; ^4^School of Medicine, College of Medicine, National Sun Yat-sen University, Kaohsiung City, Taiwan; ^5^Department of Anesthesiology, Chi Mei Medical Center, Tainan City, Taiwan; ^6^Department of Chinese Medicine, Chi Mei Medical Center, Tainan City, Taiwan; ^7^Department of Medical Imaging, Chi Mei Medical Center, Tainan City, Taiwan; ^8^Department of Health and Nutrition, Chia Nan University of Pharmacy and Science, Tainan City, Taiwan

**Keywords:** cognitive function, general anesthesia, regional anesthesia, dementia, risk factors

## Abstract

**Background:**

Dementia is a gradual and ongoing cognitive decline due to damage to nerve cells in the brain. This meta-analysis aimed to assess the potential relationship between regional anesthesia (RA) and the risk of dementia.

**Methods:**

Electronic databases including Embase, Medline, Google Scholar, and Cochrane Library were searched for studies investigating the association between RA and dementia risk from inception to March 2022. The primary outcome was the risk of dementia in patients who underwent RA (RA group) and those who received general anesthesia (GA group). Secondary outcomes included identifying other potential risk factors for dementia and comparing dementia risk between individuals receiving RA and those not receiving surgery/anesthesia (placebo group).

**Results:**

Eight cohort studies published between 2014 and 2023 were included in this analysis. A meta-analysis of the available data demonstrated no differences in baseline characteristics and morbidities (i.e., age, male proportion, hypertension, diabetes, depression, and severe comorbidities) between the RA and GA groups (all *p* > 0.05). Initial analysis revealed that the risk of dementia was higher in the GA group than in the RA group (HR = 1.81, 95% CI = 1.29–2.55, *p* = 0.007, *I*^2^ = 99%, five studies). However, when a study featuring a relatively younger population was excluded from the sensitivity analysis, the results showed a similar risk of dementia (HR, 1.17; *p* = 0.13) between the GA and RA groups. The pooled results revealed no difference in dementia risk between the RA and placebo groups (HR = 1.2, 95% CI = 0.69–2.07, *p* = 0.52, *I*^2^ = 68%, three studies). Sensitivity analysis revealed that the evidence was not stable, suggesting that limited datasets precluded strong conclusions on this outcome. Anxiety, stroke history, hypertension, diabetes, hyperlipidemia, and diabetes are potential predictors of dementia.

**Conclusion:**

Our results emphasize that, while RA could be protective against dementia risk compared to GA, the association between the type of anesthesia and dementia risk might vary among different age groups. Owing to the significant prevalence of dementia among older people and their surgical needs, further investigations are warranted to clarify the association between dementia risk and regional anesthesia.

**Systematic review registration**: https://www.crd.york.ac.uk/prospero/, CRD42023411324.

## 1 Introduction

Dementia, which is a gradual and ongoing decline in cognition due to damage to nerve cells in the brain (1), is a progressive and irreversible disease that affects a person’s ability to think, reason, communicate, and perform daily activities (2). The global incidence of dementia rises sharply with age from 5 to 7% in individuals above the age of 60–20% in those over the age of 85 years (3). As the disease progresses, patients with dementia may require assistance with daily activities and may eventually require 24-h care in a long-term care facility (4). In addition to the impact on the health and well-being on an individual level, dementia can have a significant influence on medical resources (5). Those with dementia often require frequent medical attention, including hospitalizations, emergency department visits, and specialist consultations (6–8), imposing a major public health burden worldwide. Efforts are needed to improve early detection and diagnosis as well as avoidance of related risk factors.

As the global population ages, the demand for surgical procedures is expected to increase significantly (9). In particular, orthopedic procedures, such as hip and knee replacements, are expected to rise due to aging and the corresponding increase in the prevalence of obesity (10–12). Given that the incidence of dementia tends to rise with advancing age (3), early recognition and mitigation of surgery-related risk factors for dementia in the aged population are critical. Taking into account the results of a prior meta-analysis that reported a potential association between the exposure of general anesthesia (GA) and an increased likelihood of developing dementia (13), the use of alternative anesthetic strategies may be preferred for older individuals undergoing surgery. On the other hand, compared to GA, controversy still exists over the correlation of regional anesthesia (RA) with a decreased risk of dementia in surgical populations (14, 15). Due to the lack of pooled evidence regarding the beneficial effect of RA against the risk of dementia, the objective of this meta-analysis was to assess the potential relationship between RA exposure and the risk of dementia compared with the use of GA.

## 2 Method

We registered the protocol of the current meta-analysis on PROSPERO (registration no: CRD42023411324). The report of this study followed the PRISMA criteria.

### 2.1 Data sources and search strategy

Two independent investigators conducted a search in MEDLINE, EMBASE, Cochrane Library, and Google Scholar for articles focusing on the association of RA with the long-term risk of dementia from inception to March 26, 2023. The search terms included: (“Subarachnoid block” or “Intrathecal anesthesia” or “Spinal block” or “Epidural block” or “Epidural anesthesia” or “spinal anesthesia” or “Epidural nerve block” or “Epidural spinal anesthesia” or “Lumbar epidural anesthesia” or “Lumbar epidural block” or “regional anesthesia” or “Extradural Anesthesia” or “epidural anesthesia” or “neuraxial anesthesia”) and (“General anesthesia” or “Inhalational anesthesia” or “Gas anesthesia” or “Inhalation anesthesia” or “Volatile anesthesia” or “Vapor anesthesia” or “Gaseous anesthesia” or “Inhalation general anesthesia” or “total intravenous anesthesia” or “propofol” or TIVA) and (dementia or Alzheimer’s disease). To facilitate a comprehensive search, both controlled vocabulary and synonyms were used with no restrictions on language and publication date. The search syntax for one database (i.e., MEDLINE) was summarized in Supplementary Table 1. The investigators also scanned the reference lists of the related articles to ensure the identification of all relevant studies.

### 2.2 Eligibility criteria for study inclusion

The following inclusion criteria were employed: (a) Population: adults (i.e., 18 years of age or above) without prior history of dementia undergoing surgery regardless of its type and duration; (b) Exposure: patients receiving surgery under RA served as the exposure group; (c) Control: patients subjected to surgery under GA or those did not undergo surgery/anesthesia served as control groups; (d) Outcomes: availability of data on the risk of dementia [i.e., hazard ratio (HR)]; and (e) Type of study: randomized controlled studies or cohort studies (e.g., case–control studies or population-based studies).

The exclusion criteria were (1) studies that focused on patients undergoing surgery under peripheral nerve block; (2) studies that had no information on events or the risk of dementia; (3) surgical procedures involving a combination of RA and GA; and (4) articles presented as reviews, conference abstracts, letters, case reports, or non-peer-reviewed articles.

### 2.3 Data extraction

Information relevant to the current study, namely the author, publication year, patient characteristics (e.g., age), sample size, study design, risk of dementia, severity of comorbidities, maximum follow-up time, and country of publication, was extracted. Two authors independently retrieved the data using a specific data extraction sheet. A discussion was held to resolve all disagreements. Whenever there was any uncertainty or missing information, the corresponding authors of the studies were contacted for clarification.

### 2.4 Outcomes and definitions

The main objective of this meta-analysis was to examine the likelihood of dementia in patients who underwent RA (i.e., RA group) vs. those who received GA (i.e., GA group). The secondary outcomes included identifying other potential risk factors for dementia and comparing dementia risk between individuals who received RA and those who did not undergo surgery/anesthesia (i.e., placebo group). The definition of RA for this meta-analysis encompassed spinal or epidural anesthesia with or without the use of adjunct agents such as local anesthesia and sedation, while GA involved the use of volatile or intravenous agents for anesthesia maintenance. Patients who had a Charlson comorbidity score or American Society of Anesthesiologists (ASA) physical status classification system score of 3 or higher were regarded as having severe comorbidities.

### 2.5 Risk of bias assessment and certainty of evidence

The quality of articles was assessed using the Newcastle-Ottawa Scale (NOS) that consists of eight items grouped under three categories: selection, comparability, and outcome. The selection category includes three items that examine the representativeness of the exposed and unexposed cohorts, the selection of controls, and the assessment of exposure. The comparability category has one item that evaluates the comparability of the cohorts. The outcome category consists of four items that assess the measurement of outcomes, the length and adequacy of follow-up, as well as the ascertainment of exposure. Each item is scored based on predefined criteria, with a higher score indicating a lower risk of bias. The maximum score for the NOS is nine stars. Studies with a score of seven or more stars are considered to have a “low-risk” of bias.

Two authors utilized the Grading of Recommendations Assessment, Development and Evaluation (GRADE) method (16) to evaluate the certainty of evidence for the study outcomes. For any difference in opinion between the two authors, a third author was invited to resolve the discrepancy through arbitration.

### 2.6 Statistical analysis

The data were analyzed using the random-effects model to determine the pooled odds ratio (OR) and hazard ratio (HR). Additionally, the 95% confidence interval was calculated and reported for each outcome. To check for heterogeneity, *I*^2^ statistics values ≥50% were considered to represent substantial heterogeneity. The reliability of the primary and secondary outcomes was checked by conducting a sensitivity analysis using a leave-one-out approach. Potential publication bias was identified for outcomes that were reported in 10 or more studies through a visual analysis of a funnel plot. The statistical analyses were conducted using either the Review Manager (RevMan) or comprehensive Meta-Analysis (CMA) V3 software (Biostat, Englewood, NJ, United States). A probability value (*p*) of less than 0.05 was considered to be of statistical significance.

## 3 Results

### 3.1 Study selection

**Figure 1:**
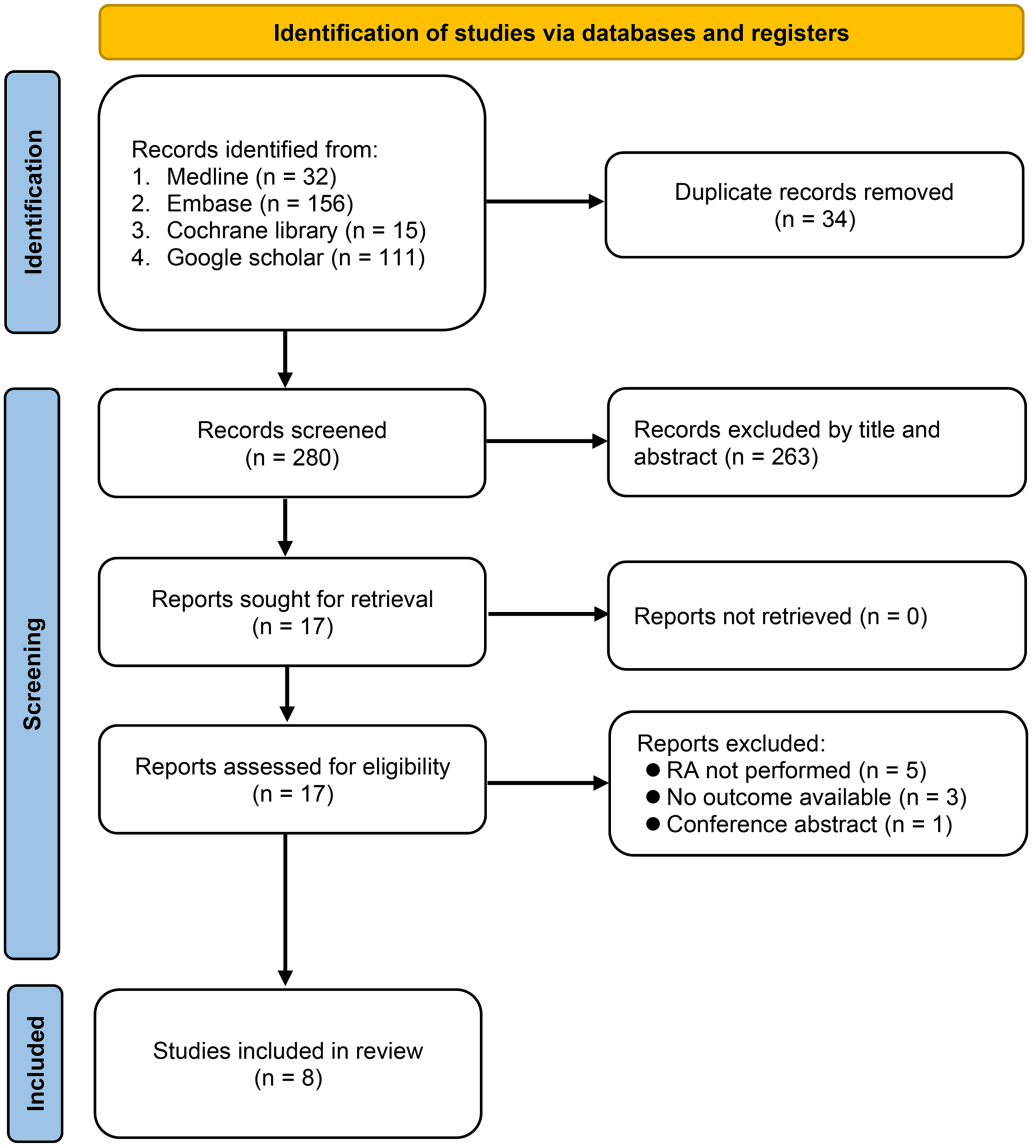
Selection process of studies based on inclusion and exclusion criteria. RA, Regional anesthesia.

Figure 1 shows the process of comprehensive search for relevant studies. We conducted searches in multiple databases, including Medline, Embase, Cochrane library, and the Google scholar, resulting in the identification of 314 potentially eligible studies. After removing duplicates (*n* = 34) and conducting title and abstract screening, 17 reports were assessed for eligibility. After further exclusion of nine studies based on full-text review due to not performing RA (*n* = 5), not reporting relevant outcomes (*n* = 3), and being a conference abstract (*n* = 1), eight cohort studies published between 2014 and 2023 were included in the final review (14, 15, 17–22).

The characteristics of studies including age, gender distribution, sample size, study design, maximum follow-up duration, and country are summarized in Table 1. The populations in the eligible studies included individuals aged 50 years and above (14), 55 years and above (19), 58 years and above (15), 65 years and above (17), 66 years and older (22), female patients aged ≥30 years undergoing hysterectomy (18), patients who received elective hip fracture surgery (20), and patients who underwent major elective surgery (21). The sample sizes varied widely, ranging from 877 to 280,308. The smallest sample size was noted in a case–control study from Sweden (i.e., 877) (15), while a population-based study from Taiwan had the largest sample size (i.e., 280,308) (18). The proportion of males in each study varied between zero and 49.5%. Regarding study design, there were five population-based studies (14, 18, 20–22), one cohort study (19), one case–control study (15), and one prospective study (17). The maximum follow-up period ranged from 5 to 20 years. The studies were conducted in various countries, including the United States (17), Taiwan (14, 18, 20, 21), Korea (19), and Canada (22).

**Table 1:** Characteristics of included studies (*n* = 8).

Study	Population	Age (years)	Male proportion	Sample size	Study design	Follow-up time (year)	Country	NOS
Aiello Bowles et al. (17)	Patients aged ≥65 years.	73 (69–79)^a^	41%^a^	3,988	Prospective study	12	United States	6
Chen et al. (14)	Patients aged ≥50 years.	63 (55–71) vs. 61 (54–69)^b^	49.5 vs. 48.2%^b^	135,873	Population-based study	3–7	Taiwan	7
Chen et al. (18)	Female patients aged ≥30 years undergoing hysterectomy.	NA	0	280,308	Population-based study	8.6	Taiwan	7
Kwon et al. (19)	Patients aged ≥55 years.	55–64 years: 42.2 vs. 42.2% 65–74 years: 35.6 vs. 35.6% ≥75 years: 22.3 vs. 35.6%^c^	45.5 vs. 45.5%^c^	5,610	Cohort study^†^	10	Korea	9
Strand et al. (15)	Patients aged ≥58 years.	82.0 ± 0.4 vs. 76.7 ± 0.4^d^	43.3 vs. 48.1%^d^	877	Case–control study	20	Sweden	6
Sun et al. (20)	Patients ≥65 years who received elective hip fracture surgery.	66.7 ± 6.4 vs. 66.3 ± 6.3 vs. 66.3 ± 6.3^e^	40 vs. 40.1 vs. 40.2%^e^	268,014	Population-based study^†^	8.22	Taiwan	9
Sun et al. (21)	Patient aged >20 years who underwent major elective surgery.	49.49 ± 16.51 vs. 49.46 ± 16.41 vs. 49.30 ± 16.55^e^	44.8 vs. 44.3 vs. 44.3%^e^	63,750	Population-based study^†^	7.2	Taiwan	9
Velkers et al. (22)	Patients aged ≥66 years.	74.3 ± 5.8 vs. 74.3 ± 5.8^f^	41.9 vs. 41.9%^f^	14,998	Population-based study^†^	5	Canada	9

^†^Matched; ^a^Data are presented as overall population; ^b^Data are presented as anesthesia vs. control group; ^c^Data are presented as RA vs. non-anesthesia group; ^d^Data are presented as dementia vs. non-dementia group; ^e^Data are presented as RA vs. inhalation vs. non-inhalation group; ^f^Data are presented as RA vs. GA group; NOS, Newcastle-Ottawa Scale.

A summary of study qualities is presented in Table 1, which indicated a variation based on NOS. Four studies (19–22) were assigned a score of nine while two studies (14, 18) were assigned a score of 7. In contrast, two others (15, 17) received a relatively low score of 6, suggesting possible limitations in terms of study quality.

### 3.2 Outcomes

#### 3.2.1 Baseline characteristics between RA and GA groups

**Figure 2:**
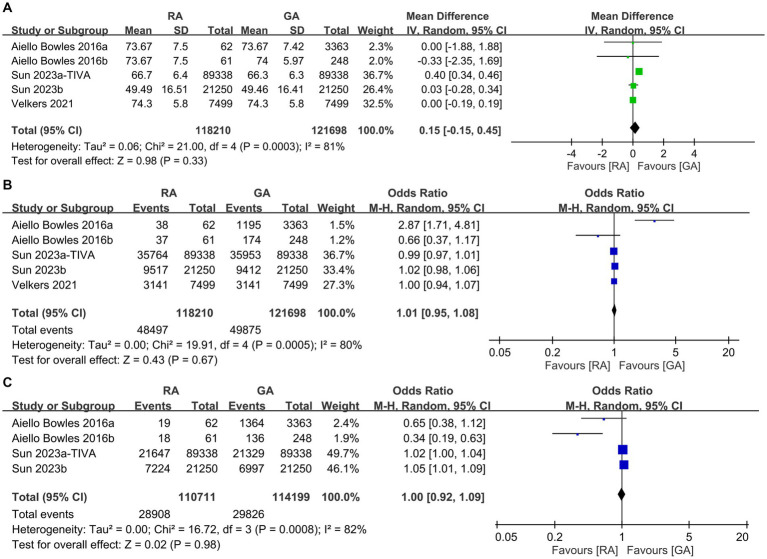
Forest plot showing the difference in **(A)** age, **(B)** male proportion, and **(C)** incidence of hypertension between individuals undergoing regional anesthesia (RA group) and general anesthesia (GA group). M-H, Mantel–Haenszel; IV, Inverse variance; and CI, Confidence interval.

**Figure 3:**
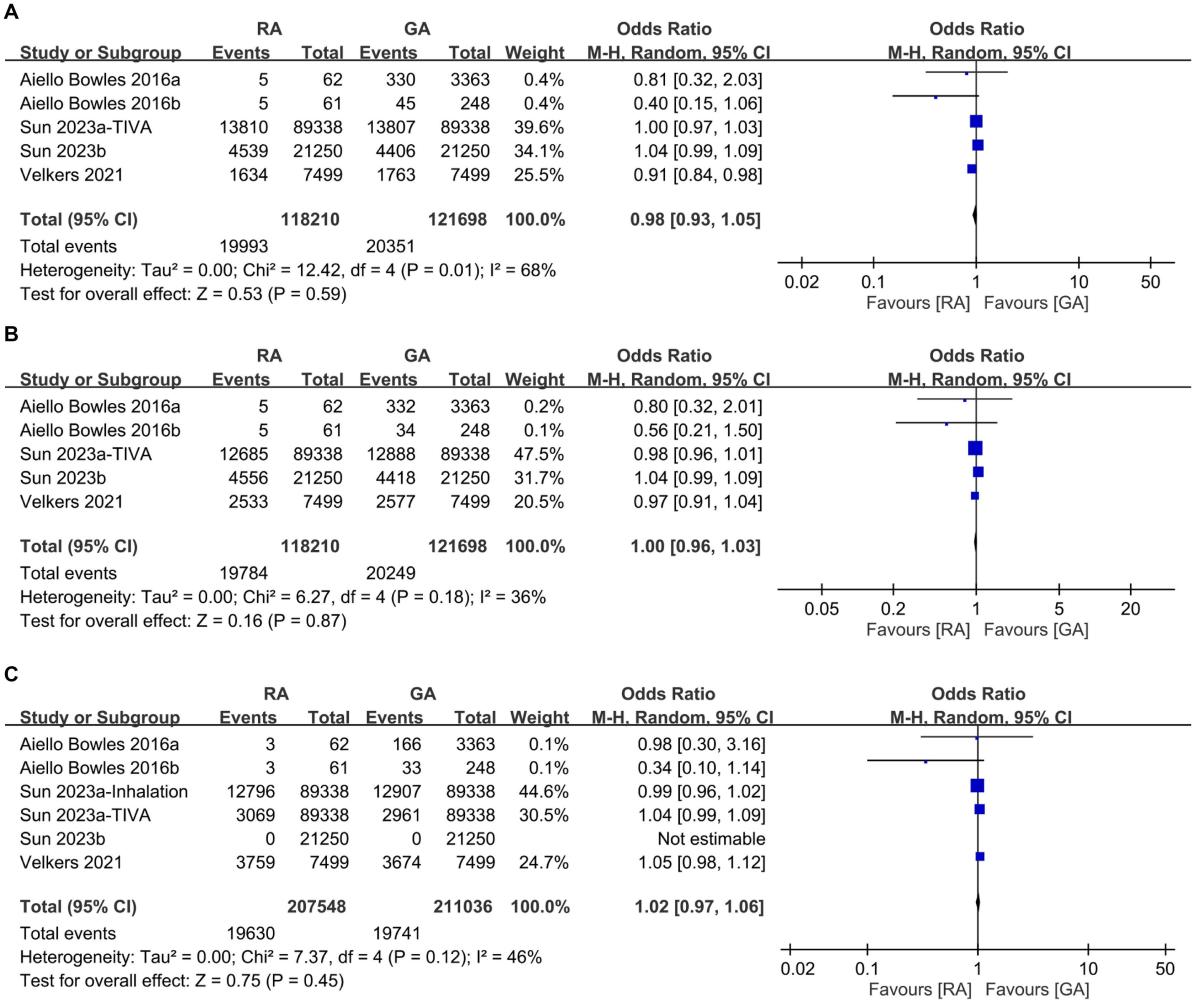
Forest plot comparing the incidence of **(A)** diabetes, **(B)** depression, and **(C)** severe comorbidities between individuals receiving regional anesthesia (RA group) and general anesthesia (GA group). M-H, Mantel–Haenszel; CI, Confidence interval.

To examine potential differences in baseline characteristics, we initially analyzed the age, incidence of male proportion, hypertension, diabetes, depression, and severity of comorbidities between the RA and GA groups. Some studies in our meta-analysis were designed to compare GA with placebo groups or RA with placebo groups. Consequently, not all studies provided the specific data required for a direct comparison between the GA and RA groups in terms of patient characteristics, such as age and sex. As shown in Figure 2, there were no differences in age (MD: 0.15 years, 95% CI: −0.15 to 0.45, *p* = 0.33, *I*^2^ = 81%) (Figure 2A), incidence of male proportion (OR: 1.01, 95% CI: 0.95–1.08, *p* = 0.67, *I*^2^ = 80%) (Figure 2B), and hypertension (OR: 1.0, 95% CI: 0.92–1.09, *p* = 0.98, *I*^2^ = 82%) (Figure 2C) between the two groups. Similarly, the incidences of diabetes (OR: 0.98, 95% CI: 0.93–1.05, *p* = 0.59, *I*^2^ = 68%) (Figure 3A), depression (OR: 1.0, 95% CI: 0.96–1.03, *p* = 0.87, *I*^2^ = 36%) (Figure 3B), and severe comorbidities (OR: 1.02, 95% CI: 0.97–1.06, *p* = 0.45, *I*^2^ = 46%) (Figure 3C) were comparable between the two groups.

#### 3.2.2 Difference in risk of dementia between RA and GA groups

**Figure 4:**
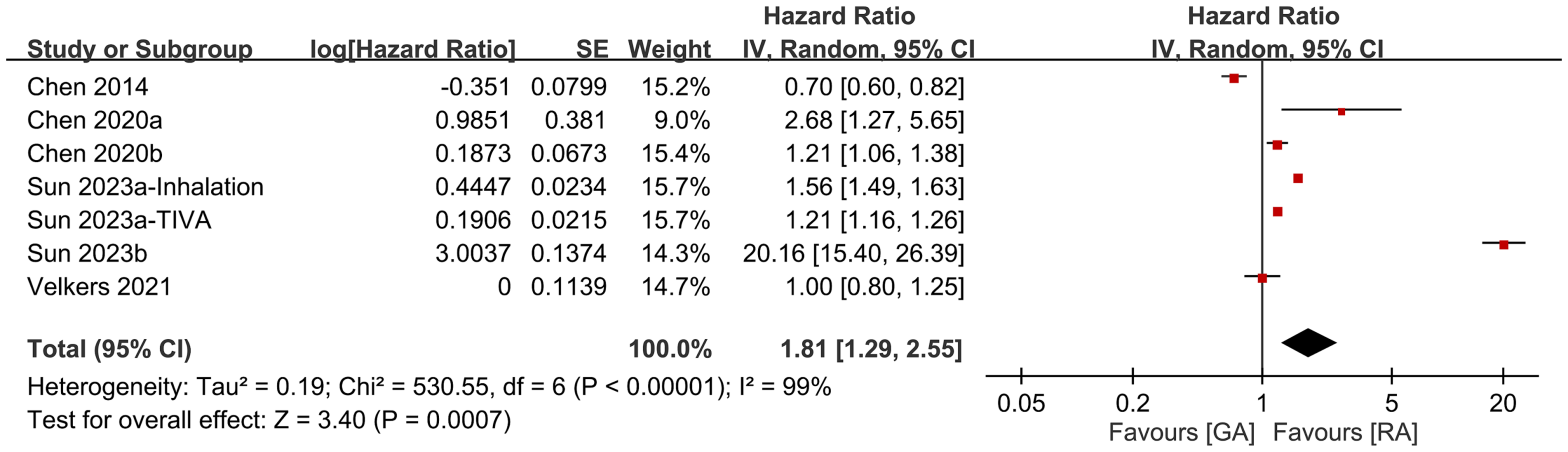
Forest plot showing the difference in risk of dementia between patients receiving regional anesthesia (RA) and those undergoing general anesthesia (GA). SE, Standard error; IV, Inverse variance; and CI, Confidence interval.

Five studies provided information for comparing the risk of dementia between patients receiving RA and those undergoing GA. Meta-analysis revealed a higher risk of dementia after GA exposure than that following RA exposure (HR: 1.81, 95% CI: 1.29–2.55, *p* = 0.007, *I*^2^ = 99%) (Figure 4) (14, 18, 20–22). When one study (21) featuring a relatively younger population was excluded from the sensitivity analysis, the results showed a notable change. The comparison between GA and regional RA exposure demonstrated a similar risk of dementia (HR, 1.17; 95% CI: 0.95–1.43, *p* = 0.13; *I*^2^ = 96%) after this exclusion. This adjusted outcome implies that in an older population, RA exposure may not offer a protective effect against dementia.

We did not examine the funnel plot as only seven datasets were available.

#### 3.2.3 Risk of dementia between RA and placebo groups

**Figure 5:**
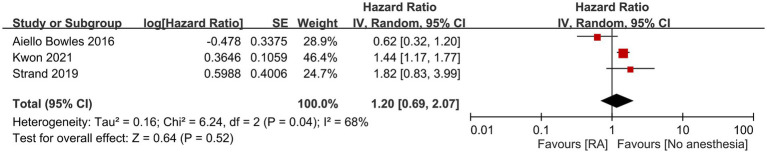
Forest plot showing the risk of dementia between regional anesthesia (RA) and placebo groups. SE, Standard error; IV, Inverse variance; and CI, Confidence interval.

Three studies provided the dementia risk in RA and placebo groups (i.e., those receiving no anesthesia/surgery). Meta-analysis revealed no difference in dementia risk between RA and placebo groups (HR: 1.2, 95% CI: 0.69–2.07, *p* = 0.52, *I*^2^ = 68%) (Figure 5) (15, 17, 19). However, when one study was removed from the pooled evidence, a higher risk of dementia was noted in the RA group, indicating questionable robustness of the pooled evidence (17).

#### 3.2.4 Other risk factors for dementia

**Figure 6:**
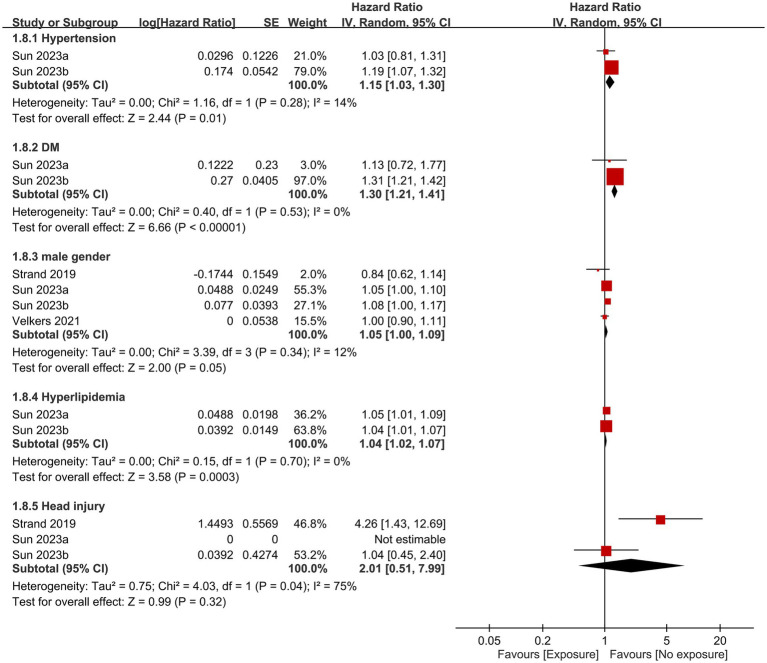
Associations of hypertension, diabetes mellitus (DM), male gender, hyperlipidemia, and head injury with the risk of dementia. SE, Standard error; IV, Inverse variance; and CI, Confidence interval.

**Figure 7:**
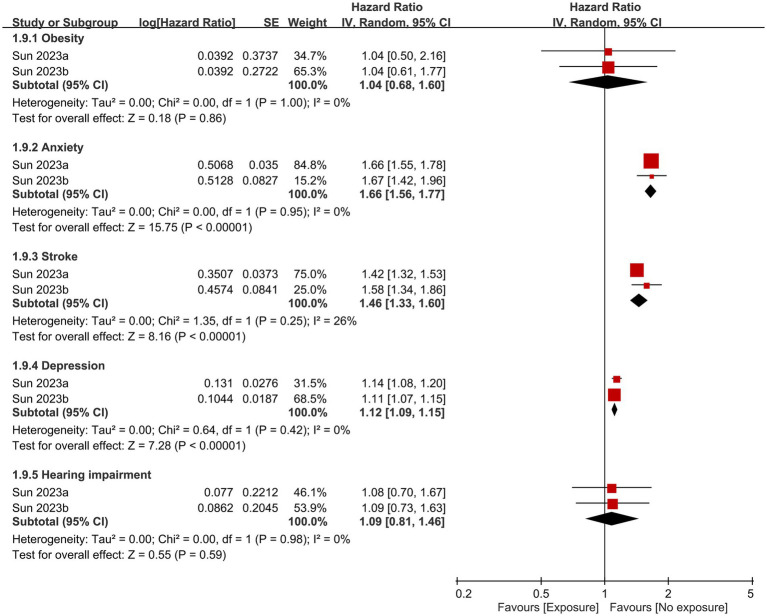
Correlations of obesity, anxiety, stroke history, depression, and hearing impairment with the risk of dementia. SE, Standard error; IV, Inverse variance; and CI, Confidence interval.

Other factors that contributed to dementia based on the current study are shown in Figures 6, 7. Anxiety, history of stroke, hypertension, diabetes, and hyperlipidemia were identified as significant risk factors for dementia. In contrast, the likelihood of developing dementia was not associated with male gender, or the presence of head injuries, obesity, and hearing impairment.

### 3.3 Certainty of evidence

The level of certainty of evidence for various outcomes is presented in Supplementary Table 2. Due to the inherent limitations of observational studies, the certainty of evidence for five outcomes, namely anxiety, history of stroke, diabetes, hyperlipidemia, and male gender, was deemed to be low. Similarly, for six other outcomes, including risk of dementia between RA and GA groups, risk of dementia between RA and placebo groups, hypertension, head injuries, obesity, and hearing impairment, the certainty of evidence was considered to be very low.

## 4 Discussion

An objective of this meta-analysis was to synthesize available evidence from previous studies to provide a better understanding of the potential association between RA exposure and dementia risk. Our findings showed a higher risk of dementia associated with GA exposure compared to RA exposure. As the pooled data did not reveal any noteworthy difference in age, male proportion, incidence of hypertension, diabetes, and depression, as well as the severity of comorbidities between patients undergoing RA versus those receiving GA, the bias of our results may be minimized. In addition, a lack of difference in dementia risk between RA and placebo groups further supported an absence of correlation between surgery under RA and the risk of dementia. Besides GA, additional risk factors for dementia were identified as hypertension, diabetes, hyperlipidemia, anxiety, history of stroke, and depression, while male gender, history of head injury, obesity, and hearing impairment were not associated with dementia risk.

Focusing on the impact of GA on short-term cognitive dysfunction, although postoperative delirium can occur in up to 28% of older person subjected to major surgery under general anesthesia (23), the link between GA and cognitive dysfunction after surgery has not been well established. For instance, one retrospective study involving older person aged over 60 who underwent major non-cardiac surgery reported no significant difference in the incidence of postoperative cognitive dysfunction (POCD) between patients undergoing GA (i.e., 14.3%) and those receiving RA (i.e., 13.9%) at the three-month follow-up (24). A recent randomized clinical trial comprising 950 older adults who underwent surgical repair for hip fracture also found that RA without sedation did not significantly reduce the incidence of postoperative delirium in comparison with GA (6.2 vs. 5.1%, respectively) (25). Consistently, a meta-analysis comprising eight studies involving 3,555 older person aged over 65 years who underwent hip-fracture surgery revealed no statistically significant difference in the prevalence of postoperative delirium (POD) or postoperative cognitive dysfunction (POCD) between those receiving RA and those undergoing GA at 24 h, 3 days, and 7 days after surgery (26), indicating a potentially important role of surgery or other provoking factors (e.g., postoperative pain) (27, 28) rather than the choice of anesthesia in the development of short-term postoperative cognitive dysfunction.

Several experimental studies have demonstrated a potential association between an exposure to inhalation agents and the neuropathogenesis of Alzheimer’s Disease (AD) as reflected by an increased production and aggregation of β-amyloid peptides (Aβ) as well as elevated levels of cerebrospinal fluid tau protein (29–32). Regarding the correlation of GA with long-term dementia risk, early evidence from clinical studies did not find a link between GA and dementia risk. For instance, there was no significant correlation between prior exposure to GA and the development of AD in a meta-analysis published in 2011 that examined 15 studies involving 1,752 cases and 5,261 controls (33). However, a subsequent population-based study including 135,873 patients showed a dose-dependent relationship between GA and dementia risk (14), suggesting that early evidence may be inconclusive. A relatively recent meta-analysis, which involved 23 studies and 412,253 participants over the age of 60 or 65 years without a pre-existing diagnosis of dementia or AD, revealed a significant positive correlation between exposure to GA and the incidence of AD despite significant heterogeneity (13). Therefore, even though a link between GA and short-term cognitive dysfunction has not been established, it appears that GA might be a potential risk factor for dementia.

Current interest in the prevention of dementia focuses on the identification of potentially modifiable risk factors (5, 34). Previous pooled evidence supported no impact of anesthetic techniques (i.e., RA vs. GA) on short-term cognitive dysfunction (26), while the influence of different anesthetic approaches on long-term dementia risk remained to be clarified. Compared with RA, our meta-analysis demonstrated a higher risk of dementia associated with exposure to GA. However, sensitivity analysis indicated that among older individuals, exposure to RA might not provide a protective effect against dementia. This discovery is significant, highlighting that older patients should employ preventive strategies irrespective of the type of anesthesia used. To the best of our knowledge, our meta-analysis is the first to address a potential beneficial effect of RA on the long-term dementia risk compared to GA. In addition, there was no significant difference in dementia risk between individuals receiving RA and the placebo group (i.e., those who did not receive surgery or anesthesia), supporting that RA may be protective against long-term dementia associated with surgery-related factors. As there is no curative treatment for patients with dementia, preventive measures are of critical importance (35). Dementia has become an increasingly costly and burdensome condition as populations age. Indeed, it is predicted that the number of patients with AD will increase to approximately 13.8 million by the year 2050 (36). Our findings showed that RA may be a favorable alternative to GA for older adults or those at high risk of dementia.

It is important to note that for some comparisons, including the analysis of dementia risk in the RA group versus placebo, only a limited number of studies were available. Although pooling results from three studies for this outcome suggested a comparable dementia risk with RA compared to placebo, sensitivity analysis revealed a contradictory finding. This highlights the unreliability of drawing conclusions from such a small number of studies. The limited datasets available for certain analyses are a major limitation of our work, which precludes strong conclusions from those particular comparisons. Larger, high-quality studies are needed to more definitively determine the impact of RA on dementia risk relative to both GA and no anesthesia exposure.

The development of dementia is influenced by a variety of factors, including environmental factors (e.g., air pollution), genetics (e.g., apolipoprotein E gene), and comorbidities (e.g., hypertension or diabetes) (37–41). In the current meta-analysis, anxiety, history of stroke, hypertension, diabetes, hyperlipidemia, and diabetes were identified as dementia risk factors. In contrast, male gender, head injuries, obesity, and hearing impairment did not increase the likelihood of developing dementia. Existing literature suggests a possible gender-related difference in dementia risk. Specifically, a previous study revealed that women exhibit a higher incidence of AD compared to men after the age of 85 (42). In contrast, another investigation reported an overall lower incidence of vascular dementia in women than that in men (43). However, it is worth noting that some studies conducted in the Italian and Spanish populations did not observe a significant association between gender and dementia risk (44, 45). In concert with the finding of those studies, the absence of a significant influence of gender on dementia risk in our study may be due to the enrollment of individuals from different ethnic backgrounds as well as the inclusion of both AD and vascular dementia in our analysis.

The results of the current meta-analysis may have several limitations that may restrict the extrapolation of our findings. First, the scarcity of existing studies may impede the strength of evidence. In addition, the relatively small sample sizes in some of the studies available for specific analyses may obscure the significance of the findings as shown in the impaired robustness of our findings on sensitivity analysis. Second, given that age is a risk factor for dementia, the inclusion of individuals with a wide range of age may impact our results. Besides, the wide variation in follow-up duration from between 3 and 7 years to as long as 20 years may influence our outcomes. Third, despite our inclusion of studies recruiting individuals of different ethnicities (i.e., United States, Taiwan, Korea, and Sweden), the relatively small number of studies still limits the extrapolation of our findings to the global population. Fourth, the meta-analytical nature of the current study precluded the exclusion of confounders that may affect the outcomes (e.g., selection bias in patient allocation). Finally, variations in the techniques of anesthesia (e.g., intravenous vs. inhalational anesthesia for GA; epidural vs. spinal anesthesia for RA) as well as the type’s dementia (e.g., Alzheimer’s disease vs. vascular dementia) could introduce heterogeneity into the current meta-analysis.

## 5 Conclusion

Our results showed an increased risk of developing dementia in individuals exposed to general anesthesia than those receiving regional anesthesia, but no significant difference in the risk of dementia between those subjected to RA and those in the placebo group (i.e., those did not receive surgery and anesthesia). Furthermore, hypertension, diabetes, hyperlipidemia, anxiety, a history of stroke, and depression were identified as risk factors for dementia, while there was no significant association of male gender, history of head injury, obesity, and hearing impairment with the risk of dementia. Further investigations are necessary to elucidate the impact of regional anesthesia on dementia risk, taking into account the high prevalence of dementia as well as the increasing need for surgery among the older person.

## Data availability statement

The original contributions presented in the study are included in the article/Supplementary material, further inquiries can be directed to the corresponding authors.

## Author contributions

I-WC: Conceptualization, Data curation, Methodology, Writing – original draft, Writing – review & editing. C-KS: Data curation, Methodology, Supervision, Writing – original draft, Writing – review & editing. J-YC: Formal analysis, Investigation, Methodology, Writing – original draft, Writing – review & editing. H-TC: Project administration, Resources, Validation, Writing – original draft, Writing – review & editing. K-ML: Writing – original draft, Writing – review & editing, Data curation, Methodology. K-CH: Data curation, Writing – original draft, Writing – review & editing, Conceptualization, Investigation, Software. C-CK: Conceptualization, Investigation, Writing – original draft, Writing – review & editing.

## References

[1] Whalley LJ Dick FD McNeill G. A life-course approach to the aetiology of late-onset dementias. Lancet Neurol. (2006) 5:87–96. doi: 10.1016/S1474-4422(05)70286-6, PMID: 16361026

[2] Arvanitakis Z Shah RC Bennett DA. Diagnosis and management of dementia. JAMA. (2019) 322:1589–99. doi: 10.1001/jama.2019.4782, PMID: 31638686 PMC7462122

[3] LoGiudice D Watson R. Dementia in older people: an update. Intern Med J. (2014) 44:1066–73. doi: 10.1111/imj.12572 25367725

[4] Ribbe MW Ljunggren G Steel K Topinková E Hawes C Ikegami N . Nursing homes in 10 nations: a comparison between countries and settings. Age Ageing. (1997) 26:3–12. doi: 10.1093/ageing/26.suppl_2.3 9464548

[5] Livingston G Huntley J Sommerlad A Ames D Ballard C Banerjee S . Dementia prevention, intervention, and care: 2020 report of the lancet commission. Lancet. (2020) 396:413–46. doi: 10.1016/S0140-6736(20)30367-6, PMID: 32738937 PMC7392084

[6] Wimo A Jönsson L Gustavsson A McDaid D Ersek K Georges J . The economic impact of dementia in Europe in 2008—cost estimates from the Eurocode project. Int J Geriatr Psychiatry. (2011) 26:825–32. doi: 10.1002/gps.2610 21744385

[7] Williamson LE Evans CJ Cripps RL Leniz J Yorganci E Sleeman KE. Factors associated with emergency department visits by people with dementia near the end of life: a systematic review. J Am Med Dir Assoc. (2021) 22:2046–2055.e35. doi: 10.1016/j.jamda.2021.06.012 34273269

[8] Benner M Steiner V Pierce LL. Family caregivers’ reports of hospitalizations and emergency department visits in community-dwelling individuals with dementia. Dementia. (2018) 17:585–95. doi: 10.1177/1471301216653537, PMID: 29968510

[9] Etzioni DA Liu JH Maggard MA Ko CY. The aging population and its impact on the surgery workforce. Ann Surg. (2003) 238:170–7. doi: 10.1097/01.SLA.0000081085.98792.3d, PMID: 12894008 PMC1422682

[10] Harms S Larson R Sahmoun A Beal J. Obesity increases the likelihood of total joint replacement surgery among younger adults. Int Orthop. (2007) 31:23–6. doi: 10.1007/s00264-006-0130-y 16688455 PMC2267551

[11] Wells V Hearn T McCaul K Anderton S Wigg A Graves S. Changing incidence of primary total hip arthroplasty and total knee arthroplasty for primary osteoarthritis. J Arthroplast. (2002) 17:267–73. doi: 10.1054/arth.2002.30414 11938500

[12] Pilz V Hanstein T Skripitz R. Projections of primary hip arthroplasty in Germany until 2040. Acta Orthop. (2018) 89:308–13. doi: 10.1080/17453674.2018.1446463, PMID: 29504824 PMC6055773

[13] Lee JJ Choi GJ Kang H Baek CW Jung YH Shin HY . Relationship between surgery under general anesthesia and the development of dementia: a systematic review and meta-analysis. Biomed Res Int. (2020) 2020:1–22. doi: 10.1155/2020/3234013 PMC7165327 32337238

[14] Chen PL Yang CW Tseng YK Sun WZ Wang JL Wang SJ . Risk of dementia after anaesthesia and surgery. Br J Psychiatry. (2014) 204:188–93. doi: 10.1192/bjp.bp.112.119610, PMID: 23887997 PMC3939441

[15] Strand AK Nyqvist F Ekdahl A Wingren G Eintrei C. Is there a relationship between anaesthesia and dementia? Acta Anaesthesiol Scand. (2019) 63:440–7. doi: 10.1111/aas.13302, PMID: 30511411

[16] Guyatt GH Oxman AD Vist GE Kunz R Falck-Ytter Y Alonso-Coello P . GRADE: an emerging consensus on rating quality of evidence and strength of recommendations. BMJ. (2008) 336:924–6. doi: 10.1136/bmj.39489.470347.AD, PMID: 18436948 PMC2335261

[17] Aiello Bowles EJ Larson EB Pong RP Walker RL Anderson ML Yu O . Anesthesia exposure and risk of dementia and Alzheimer's disease: a prospective study. J Am Geriatr Soc. (2016) 64:602–7. doi: 10.1111/jgs.14024, PMID: 26865152 PMC4803643

[18] Chen YC Oyang YJ Lin TY Sun WZ. Risk assessment of dementia after hysterectomy: analysis of 14-year data from the National Health Insurance Research Database in Taiwan. J Chin Med Assoc. (2020) 83:394–9. doi: 10.1097/JCMA.0000000000000286, PMID: 32149891 PMC13048180

[19] Kwon Y-S Lee J-J Lee S-H Kim C Yu H Sohn J-H . Risk of dementia in patients who underwent surgery under neuraxial anesthesia: a nationwide cohort study. J Personal Med. (2021) 11:1386. doi: 10.3390/jpm11121386, PMID: 34945858 PMC8708516

[20] Sun M Chen WM Wu SY Zhang J. Dementia risk amongst older adults with hip fracture receiving general anaesthesia or regional anaesthesia: a propensity-score-matched population-based cohort study. Br J Anaesth. (2023) 130:305–13. doi: 10.1016/j.bja.2022.11.014, PMID: 36593163

[21] Sun M Chen WM Wu SY Zhang J. Dementia risk after major elective surgery based on the route of anaesthesia: a propensity score-matched population-based cohort study. EClinicalMedicine. (2023) 55:101727. doi: 10.1016/j.eclinm.2022.101727, PMID: 36386032 PMC9641180

[22] Velkers C Berger M Gill SS Eckenhoff R Stuart H Whitehead M . Association between exposure to general versus regional anesthesia and risk of dementia in older adults. J Am Geriatr Soc. (2021) 69:58–67. doi: 10.1111/jgs.16834, PMID: 33025584 PMC7942805

[23] Evered LA Chan MT Han R Chu MH Cheng BP Scott DA . Anaesthetic depth and delirium after major surgery: a randomised clinical trial. Br J Anaesth. (2021) 127:704–12. doi: 10.1016/j.bja.2021.07.021 34465469 PMC8579421

[24] Rasmussen LS Johnson T Kuipers HM Kristensen D Siersma VD Vila P . Does anaesthesia cause postoperative cognitive dysfunction? A randomised study of regional versus general anaesthesia in 438 elderly patients. Acta Anaesthesiol Scand. (2003) 47:260–6. doi: 10.1034/j.1399-6576.2003.00057.x, PMID: 12648190

[25] Li T Li J Yuan L Wu J Jiang C Daniels J . Effect of regional vs general anesthesia on incidence of postoperative delirium in older patients undergoing hip fracture surgery: the RAGA randomized trial. JAMA. (2022) 327:50–8. doi: 10.1001/jama.2021.22647, PMID: 34928310 PMC8689436

[26] Bhushan S Huang X Duan Y Xiao Z. The impact of regional versus general anesthesia on postoperative neurocognitive outcomes in elderly patients undergoing hip fracture surgery: a systematic review and meta-analysis. Int J Surg. (2022) 105:106854. doi: 10.1016/j.ijsu.2022.106854, PMID: 36031067

[27] Lynch EP Lazor MA Gellis JE Orav J Goldman L Marcantonio ER. The impact of postoperative pain on the development of postoperative delirium. Anesth Analg. (1998) 86:781–5. PMID: 9539601 10.1097/00000539-199804000-00019

[28] Vaurio LE Sands LP Wang Y Mullen EA Leung JM. Postoperative delirium: the importance of pain and pain management. Anesth Analg. (2006) 102:1267–73. doi: 10.1213/01.ane.0000199156.59226.af 16551935

[29] Dong Y Zhang G Zhang B Moir RD Xia W Marcantonio ER . The common inhalational anesthetic sevoflurane induces apoptosis and increases beta-amyloid protein levels. Arch Neurol. (2009) 66:620–31. doi: 10.1001/archneurol.2009.48, PMID: 19433662 PMC2748878

[30] Xie Z Dong Y Maeda U Alfille P Culley DJ Crosby G . The common inhalation anesthetic isoflurane induces apoptosis and increases amyloid beta protein levels. Anesthesiology. (2006) 104:988–94. doi: 10.1097/00000542-200605000-00015, PMID: 16645451

[31] Eckenhoff RG Johansson JS Wei H Carnini A Kang B Wei W . Inhaled anesthetic enhancement of amyloid-beta oligomerization and cytotoxicity. Anesthesiology. (2004) 101:703–9. doi: 10.1097/00000542-200409000-00019, PMID: 15329595

[32] Perucho J Rubio I Casarejos MJ Gomez A Rodriguez-Navarro JA Solano RM . Anesthesia with isoflurane increases amyloid pathology in mice models of Alzheimer's disease. J Alzheimer's Dis. (2010) 19:1245–57. doi: 10.3233/JAD-2010-1318 20308791

[33] Seitz DP Shah PS Herrmann N Beyene J Siddiqui N. Exposure to general anesthesia and risk of Alzheimer's disease: a systematic review and meta-analysis. BMC Geriatr. (2011) 11:83. doi: 10.1186/1471-2318-11-83 22168260 PMC3258207

[34] Barnes DE Yaffe K. The projected effect of risk factor reduction on Alzheimer's disease prevalence. Lancet Neurol. (2011) 10:819–28. doi: 10.1016/S1474-4422(11)70072-2, PMID: 21775213 PMC3647614

[35] Sabayan B Sorond F. Reducing risk of dementia in older age. JAMA. (2017) 317:2028. doi: 10.1001/jama.2017.2247 28510681

[36] Hebert LE Weuve J Scherr PA Evans DA. Alzheimer disease in the United States (2010-2050) estimated using the 2010 census. Neurology. (2013) 80:1778–83. doi: 10.1212/WNL.0b013e31828726f5, PMID: 23390181 PMC3719424

[37] Alzheimer's Association. 2023 Alzheimer's disease facts and figures. Alzheimers Dement. (2023) 19:1598–1695. doi: 10.1002/alz.13016 36918389

[38] Chen Q Wang T Kang D Chen L. Protective effect of apolipoprotein E epsilon 3 on sporadic Alzheimer's disease in the Chinese population: a meta-analysis. Sci Rep. (2022) 12:13620. doi: 10.1038/s41598-022-18033-x, PMID: 35948759 PMC9365782

[39] Chen J-H Lin K-P Chen Y-C. Risk factors for dementia. J Formos Med Assoc. (2009) 108:754–64. doi: 10.1016/S0929-6646(09)60402-2 19864195

[40] Abolhasani E Hachinski V Ghazaleh N Azarpazhooh MR Mokhber N Martin J. Air pollution and incidence of dementia: a systematic review and meta-analysis. Neurology. (2023) 100:e242–54. doi: 10.1212/WNL.0000000000201419 36288998

[41] Agarwal R Tripathi CB. Association of apolipoprotein E genetic variation in Alzheimer's disease in Indian population: a meta-analysis. Am J Alzheimers Dis Other Dement. (2014) 29:575–82. doi: 10.1177/1533317514531443, PMID: 25551132 PMC10852589

[42] Andersen K Launer LJ Dewey ME Letenneur L Ott A Copeland JR . Gender differences in the incidence of AD and vascular dementia: the EURODEM studies. EURODEM incidence research group. Neurology. (1999) 53:1992–7. doi: 10.1212/WNL.53.9.1992 10599770

[43] Ruitenberg A Ott A van Swieten JC Hofman A Breteler MM. Incidence of dementia: does gender make a difference? Neurobiol Aging. (2001) 22:575–80. doi: 10.1016/S0197-4580(01)00231-7 11445258

[44] López-Pousa S Vilalta-Franch J Llinàs-Regla J Garre-Olmo J Román GC. Incidence of dementia in a rural community in Spain: the Girona cohort study. Neuroepidemiology. (2004) 23:170–7. doi: 10.1159/000078502 15272219

[45] Ravaglia G Forti P Maioli F Martelli M Servadei L Brunetti N . Incidence and etiology of dementia in a large elderly Italian population. Neurology. (2005) 64:1525–30. doi: 10.1212/01.WNL.0000160107.02316.BF, PMID: 15883312

