# Supplementary material for: Comparison of regional vs. general anesthesia on the risk of dementia: a systematic review and meta-analysis
Source: Front Public Health. 2024 Jun 3;12:1362461. doi: 10.3389/fpubh.2024.1362461 (PMC11182446; doi:10.3389/fpubh.2024.1362461)
Supplement: Supplementary file 1 [file Table_1.DOCX]

Supplemental Table 1. Search strategies for Medline

| Database | # | Search syntax |
| --- | --- | --- |
| **MEDLINE (Ovid)** | 1 | ("Subarachnoid block" or "Intrathecal anesthesia" or "Spinal block" or "Epidural block" or "Epidural aesthesia" or "spinal anesthesia" or "Epidural nerve block" or "Epidural spinal anesthesia" or "Lumbar epidural anesthesia" or "Lumbar epidural block." or "regional anesthesia" or "Extradural Anesthesia" or "epidural anesthesia").mp |
|  | 2 | exp "Anesthesia, Conduction"/ or exp "Anesthesia, Spinal"/ or exp "Anesthesia, Epidural"/ |
|  | 3 | ("General anesthesia" or "Inhalational anesthesia" or "Gas anesthesia" or "Inhalation anesthesia" or "Volatile anesthesia" or "Vapor anesthesia" or "Gaseous anesthesia" or "Inhalation general anesthesia" or "total intravenous anesthesia" or "propofol" or TIVA).mp |
|  | 4 | exp "Anesthesia, General"/ |
|  | 5 | ("dementia" or "Alzheimer's disease").mp |
|  | 6 | exp "dementia"/ or exp "Alzheimer Disease"/ |
|  | 7 | (1 OR 2) AND (3 OR 4) AND (5 OR 6) |

**Supplemental Table 2.** Summary of findings for the main comparison

| Outcomes | Relative effect (95% CI) | № of  studies | Certainty of the evidence (GRADE)‡ | Comments |
| --- | --- | --- | --- | --- |
|  |  |  |  |  |
| Risk of dementia between RA and GA groups | HR 1.81 (1.29 to 2.55) | 5 studies | ⨁◯◯◯ Very Low | b |
| Risk of dementia between RA and placebo groups | HR 1.2  (0.69 to 2.07) | 3 studies | ⨁◯◯◯ Very low | a,b |
| Anxiety | HR 1.66  (1.56 to 1.77) | 2 studies | ⨁⨁◯◯ Low | - |
| History of stroke | HR 1.46  (1.33 to 1.6) | 2 studies | ⨁⨁◯◯ Low | - |
| Hypertension | HR 1.15  (1.03 to 1.3) | 2 studies | ⨁◯◯◯ Very low | a |
| Diabetes | HR 1.3  (1.21 to 1.41) | 2 studies | ⨁⨁◯◯ Low | - |
| Hyperlipidemia | HR 1.04  (1.02 to 1.07) | 2 studies | ⨁⨁◯◯ Low | - |
| Male gender | HR 1.05  (1.0 to 1.09) | 4 studies | ⨁⨁◯◯ Low | - |
| Head injuries | HR 2.01  (0.51 to 7.99) | 3 studies | ⨁◯◯◯ Very low | a,b |
| Obesity | HR 1.04  (0.68 to 1.6) | 2 studies | ⨁◯◯◯ Very low | a |
| Hearing impairment | HR 1.09  (0.81 to 1.46) | 2 studies | ⨁◯◯◯ Very low | a |

Comments:

^a^wide 95% CI; ^b^The I square is more than 50%.

‡evidence based on observational studies starts with a low level

**GRADE Working Group grades of evidence:**
High certainty: We are very confident that the true effect lies close to that of the estimate of the effect
Moderate certainty: We are moderately confident in the effect estimate: The true effect is likely to be close to the estimate of the effect, but there is a possibility that it is substantially different
Low certainty: Our confidence in the effect estimate is limited: The true effect may be substantially different from the estimate of the effect
Very low certainty: We have very little confidence in the effect estimate: The true effect is likely to be substantially different from the estimate of effect
